# Autism Spectrum Disorder Phenotypes Based on Sleep Dimensions and Core Autism Symptoms

**DOI:** 10.1007/s10803-025-06822-y

**Published:** 2025-04-17

**Authors:** Kristina P. Lenker, Yanling Li, Julio Fernandez-Mendoza, Susan D. Mayes, Susan L. Calhoun

**Affiliations:** 1 Sleep Research & Treatment Center, Penn State Milton S. Hershey Medical Center, College of Medicine, Department of Psychiatry and Behavioral Health, Penn State University, Hershey, PA USA; 2 Social Science Research Institute, Pennsylvania State University, Hershey, PA USA; 3 Department of Psychiatry and Behavioral Health, College of Medicine, Pennsylvania State University, Hershey, PA USA

**Keywords:** Autism, Sleep, Phenotype, Disturbed sleep, Insufficient sleep, Hypersomnolence

## Abstract

Previous studies have used cluster analysis to address the diagnostic heterogeneity of autism spectrum disorder, but have been limited by identifying subgroups solely on the basis of core autism symptoms. The present study aimed to identify sleep phenotypes and their clustering with core autism symptoms in youth diagnosed with autism. 1397 patients (1–17y, M = 6.1 ± 3.3y; M IQ = 88.5 ± 27.2; 81.2% male, 89.0% white) with autism. Principal component analysis (PCA) was performed on 10 sleep items from the Pediatric Behavior Scale. Latent class analyses (LCA) determined phenotypes characterized by core autism symptoms and sleep clusters, accounting for age, sex, Intelligence Quotient (IQ), and medication use.PCA identified three distinct sleep clusters (disturbed sleep, insufficient sleep and hypersomnolence) explaining 48.4% of the variance. LCA revealed four phenotypes based on core ASD symptoms and sleep clusters. Compared to Class 1 (54.8%) as the reference group, Class 2 (26.3%) had a similar degree of sleep problems, higher IQ and milder autism symptoms, less problems with selective attention/fearlessness; Class 3 (14.5%) was characterized by insufficient and disturbed sleep, perseveration and somatosensory disturbance, and higher medication use, while Class 4 (4.4%) was by hypersomnolence, problems with social interactions, and higher medication use.We found four distinct clustering of core autism symptoms and sleep problems differing in their sleep profiles as well as in relation to clinical characteristics, demographics, internalizing/externalizing symptoms, and functional outcomes. Our findings underscore the heterogeneity of autism based on sleep-wake problems, advocating for personalized therapeutic interventions targeting nighttime sleep and daytime alertness.

**Supplementary Information:**

The online version contains supplementary material available at 10.1007/s10803-025-06822-y.

Autism Spectrum Disorder (ASD) is a lifelong neurodevelopmental disorder that manifests as core difficulty with social communication as well as restricted or repetitive and stereotyped behaviors and interests as per the Diagnostic and Statistical manual of Mental Disorders 5th Edition (APA, 2013) that significantly impacts social, educational, and emotional functioning. According to the Center for Disease Control, approximately 1 in 36 children in the United States (US) are diagnosed with ASD each year, making ASD the second most frequently diagnosed developmental disability in the US (Maenner, 2023). In addition to the core symptoms of ASD, sleep problems are a primary co-occurring concern reported by autistic individuals that impact their daytime functioning and overall quality of life, often persist into adulthood, and is an area of health often overlooked in routine medical care (Johnson & Zarrinnegar, 2021). When compared to autistic youth without sleep problems, those with ASD and sleep problems tend to have more severe symptoms of sensory disturbance, poorer social interactions, hyperactivity, and anxiety and depression, suggesting that treatment of sleep problems may contribute to the reduction of symptoms associated with ASD (Goldman et al., 2009). Children’s sleep problems also impact the entire family and often result in increased parental stress and decreased well-being (Hoffman et al., 2008). Most studies have found that difficulties falling asleep and night waking are the most common types of sleep disturbance in children with ASD (Mayes & Calhoun, 2009; Richdale & Schreck, 2009). They are known to both be exacerbated and influenced by core ASD symptomatology, co-occurring behavioral problems, and psychiatric conditions.

Core ASD symptoms and difficulties with executive functioning may hinder the acquisition of good sleep hygiene behaviors. For example, ASD-related restricted interests and repetitive behaviors may manifest as cognitive and behavioral inflexibility that results in over-reliance on rigid bedtime routines and rituals or difficulty transitioning to bed in lieu of preferred activities, exacerbating sleep problems such as difficulty falling asleep (Reynolds & Malow, 2011). ASD-related social and communication deficits may also impair a child’s understanding of what behaviors are expected and why maintaining a consistent bedtime routine is essential for adequate sleep health and the ability to meet the demands of daily living (i.e., adaptive functioning), which have been shown to predict sleep onset delay (Masi et al., 2022) and shorter sleep duration (Veatch et al., 2017). Moreover, the prevalence of sensory problems in autistic children is estimated at 69–95% in comparison to 6–14% in the general population (Baranek et al., 2006). Research has suggested that over responsivity to sensory input may play a role in the etiology of sleep problems in children with ASD (Mazurek & Petroski, 2015; Manelis-Baram et al., 2021; Holloway, 2013). In this case, children may be especially sensitive to the effects of and have difficulty filtering out stimuli within their sleep environment. Indeed, sensory hypersensitivity may contribute to hyperarousal, which is one of several mechanism that are hypothesized to exacerbate sleep problems in autistic children and cause insomnia in the general population or hyper-reactivity and unusual sensory interests such as such as excessive smelling or chewing objects (Ausderau et al., 2014; Deliens & Peigneux, 2019), and hypersensitivity/hyper-reactivity in touch, vestibular, and oral domains (Kosaka et al., 2021; Tzischinsky et al., 2018). A better understanding of the inherent autism-related characteristics linked to comorbid sleep problems would improve comprehensive assessment and lead to the development of novel treatments to ameliorate the significant and debilitating effects of these prevalent problems on children and their families.

Some studies have indicated that age plays a significant role in predicting sleep issues. According to Mayes and colleagues (2022) and Giannotti and colleagues (2008), young children tend to experience more sleep disturbances. Conversely, other research suggests that sleep problems persist in individuals with ASD and can vary across different age groups (Goldman et al., 2012; Hodge et al., 2014; Wiggs & Stores, 2004). For instance, Goldman and colleagues (2012) conducted a study involving 1859 autistic children, ranging from 3 to 18 years old, and found that sleep issues persist from early childhood through adolescence. Furthermore, their findings indicated that the nature of these problems tends to change with age. Specifically, parents of younger children reported more instances of sleep anxiety, bedtime resistance, night awakenings, and parasomnias, while parents of adolescents noted more challenges related to sleep onset, duration, and daytime sleepiness.

Prior research has consistently shown that sleep problems are associated with core ASD symptoms, symptom severity, challenging behaviors, attention problems, and adaptive functioning. Previous studies have used cluster analysis to clarify diagnostic heterogeneity of ASD, which have identified between one and four clusters, differentiated by ASD symptom severity, and labelling these subtypes as mild, moderate, and severe ASD (Syriopoulou-Deli & Papaefstathiou, 2020). However, these studies have been limited to identifying subgroups of ASD on the basis of core symptoms rather than sleep problems. Larger scale studies involving cluster analysis may help identify groups with clinically similar presentations. Furthermore, limited research has begun to consider sleep problems as a phenotypic marker, however, specific sleep phenotypes and their relationship to ASD symptoms have yet to be identified. Previous research has primarily focused on dichotomizing sleep into “good/poor sleep” (Goldman et al., 2009) and “stable/unstable sleepers” (Cohen et al., 2017) instead of encompassing the full range of sleep problems children and adolescents with ASD typically experience. Therefore, it remains unknown whether autism phenotypes can be determined based on multiple sleep problems. Moreover, there is a lack of understanding about the relationship between core ASD symptoms and sleep problems in children and adolescents with ASD.

The aims of the current study are to (1) identify specific sleep dimensions using the parent-reported Pediatric Behavior Scale (PBS) in autistic children and adolescents via principal component analysis (PCA); (2) identify distinct clusters (phenotypes) based on the PCA-identified sleep dimensions and parent-reported core ASD symptoms on the Checklist for Autism Spectrum Disorder (CASD) while accounting for age, sex, intelligence quotient (IQ), race, socio-economic status (SES) and medication use, in autistic children and adolescents via latent class analysis (LCA); and (3) contrast the demographic and clinical characteristics across the LCA-derived phenotypes based on PBS and CASD dimensions. The aims of this study are a critical step in addressing the heterogeneity of ASD by relying on a critical dimension, sleep-wake problems, typically neglected in phenotyping efforts based solely on core autism symptoms.

## Methods

### Study Sample

The current study utilized a pre-existing data set of children and adolescents evaluated at an outpatient psychiatry diagnostic clinic between May 1990 and July 2010. Youths were referred to the clinic by a primary care provider, intervention providers, schools, and/or parents because of possible ASD, ADHD, or significant behavior problems. The clinic serves rural, suburban, and urban areas in central Pennsylvania. The study was approved by the Institutional Review Board, which waived informed consent because analyses were conducted retrospectively on existing, de-identified clinical data.

The current study sample included those 1397 children diagnosed with ASD by a licensed PhD psychologist, 1–17 years of age (M = 6.1, SD = 3.3) with Full Scale IQs of 9–146 (M = 88.5, SD = 27.2), of whom 81.2% of the children are male, 89.0% are white, and 34.1% have a parent with a professional or managerial occupation. Diagnoses were based on comprehensive tests and procedures (Calhoun et al., 2017; Conrad et al., 2010; Mayes et al., 2008, 2009a, b, 2011, 2012; Waxmonsky et al., 2017), including: (1) a structured diagnostic interview with the parent after having completed the CASD (Mayes, 2012), (2) parent and teacher questionnaires and rating scales, including the PBS (Lindgren, & Koeppl, 1987), (3) child interview and self-report scales, when applicable, (4) clinical observations of the child, (5) review of the child’s developmental history, educational records from kindergarten to the present, and previous evaluations, and (6) psychological testing (IQ, achievement, attention, and neuropsychological), and (7) clinical observations during the psychological evaluation. Age, sex assigned at birth, race/ethnicity (nonminority vs. minority), SES (professional/managerial occupation vs. nonprofessional occupation), presence of pregnancy complications, and difficult infant and preschool temperament, and medication use were collected from the parent at the time of the evaluation.

### Objective Neurocognitive Testing

Different intelligence tests were administered by the licensed psychologist to cover the mental age range of the children. Tests were administered using the current version at the time the child was evaluated. For children functioning at ages 1 to 2.5 year, the Bayley Scales of Infant Development I or II was administered (Whatley, 1987). The Wechsler Preschool and Primary Scale of Intelligence III or IV was administered to children ages 2:6 to 5:11 years (Wahlstrom et al., 2012), and the Wechsler Intelligence Scale for Children–III or IV or Stanford-Binet Intelligence Scale IV was given to children functioning at 6 years or above (Peterson et al., 2020; Wahlstrom et al., 2012).

For children tested on the WISC-III, the Wechsler Individual Achievement Test (WIAT) (Wechsler, 2012) subtests were administered, including Reading Comprehension, Word Reading, Numerical Operations, and Written Expression (the last for children 8 years and older only). The WIAT Written Expression subtest requires children to write a letter describing their ideal house, which is scored for ideas and development, organization, vocabulary, sentence structure, grammar, punctuation, and capitalization.

### Parent-Reported Testing

Parents rated their children on the 165-item PBS (Lindgren, & Koeppl, 1987) on a 4-point scale (0 = almost never or not at all, 1 = sometimes, 2 = often, and 3 = very often a problem). The PBS assesses multiple psychological, developmental, and somatic problems including norm referenced T-scores for subscales for sleep problems, ADHD (hyperactivity, impulsivity, and inattention), depression, and anxiety. Scores on the PBS correspond well with the Child Behavior Checklist and objective measures of ADHD (Mayes et al., 2014). The PBS has high internal consistency for subscale scores (median coefficient is 0.91) and corresponds well with established measures of psychopathology (Mayes et al., 2014) and significantly differentiates diagnostic groups and assess sleep and psychological problems in several published studies (Calhoun et al., 2017; Conrad et al., 2010; Mayes et al., 2008, 2009a, b, 2011, 2012; Waxmonsky et al., 2017). The PBS total sleep problems score correlates highly with scores on the Children’s Sleep-Wake Scale and on the Pediatric Sleep Questionnaire, with correlations of 0.76 and 0.72 (Mayes et al., 2008). The PBS sleep disturbance score is based on seven sleep items: (1) difficulty falling asleep, (2) wakes often during the night, (3) restless during sleep, (4) nightmares, (5) walks or talks in sleep, (6) wakes too early, (7) sleeps less than most children. Three additional items related to sleep are also rated by parents: (1) sleeps more than most children, (2) sleepy during the day, and (3) drowsy during the day. Thus, these 10 items were included in the analyses.

Parents rated their children on the 30-item CASD (Mayes, 2012). The CASD is a comprehensive 30-item diagnostic checklist scored as present (either currently or in the past) or absent that is normed and standardized on 2,469 youth (1–18 years, IQs 9-146) with autism, other clinical disorders, and typical development (Mayes, 2012). In the national standardization study, the CASD differentiated youth with and without autism with 99.5% accuracy. The CASD is unique in that it was developed to describe a broad range of symptoms displayed by youth with ASD. The CASD provides detailed information on six domains associated with ASD that include (1) problems with social interactions (e.g., social isolation, self-absorbed, poor social reasoning), (2) perseveration (e.g., obsessive fixations, repetitive play, upset with change, stereotypies), (3) somatosensory disturbance (e.g., hypersensitivity to sounds, abnormal sensory inspection, tactile defensiveness), (4) atypical communication and development, as well as associated symptoms of (5) mood disturbance and (6) problems with attention and safety. Studies demonstrate that the CASD distinguishes youth with autism from youth with intellectual disability, learning disability, traumatic brain injury, language disorder, ADHD, ODD, and anxiety disorder (Mayes, 2012), apraxia of speech (Tierney et al., 2015), and reactive attachment disorder (Mayes et al., 2017). Concurrent validity is strong and studies demonstrate high diagnostic agreement between the CASD and the Childhood Autism Rating Scale (98%), the Gilliam Asperger’s Disorder Scale (94%), and the Autism Diagnostic Interview-R (93%) (Mayes et al., 2009a, b; Murray et al., 2011). All 1397 youth in the sample had received a DSM-IV or DSM-5 diagnosis of autism (i.e., autistic disorder, Asperger’s disorder, or autism spectrum disorder) using the DSM version that was current at the time the child was evaluated, and all youth had a score on the CASD in the autism range, i.e., 15 points or greater (Mayes, 2012). Youth with a diagnosis of Pervasive Developmental Disorder-Not Otherwise Specified (PD-NOS) were not included in the study sample.

### Statistical Analyses

First, to identify specific sleep dimensions in this sample of youth with autism, PCA was conducted on the 10 sleep items of the PBS. The number of dimensions retained in PCA with Varimax rotation and Kaiser normalization was determined based on eigenvalues in the scree plot. We used oblimin rotation and maximum likelihood to estimate factor loadings. A cutoff value of 0.4 was set for determining the saliency of factor loadings for each item.

Second, to identify clusters/phenotypes based on the PCA-derived sleep dimensions and CASD-assessed autism dimensions, LCA (McCutcheon, 1987; Hagenaars & McCutcheon, 2002) was used for group identification and classification. In the present study, the parameters of LCA were estimated using the maximum likelihood estimation with robust standard errors. If the percentage of individuals in a specific category was less than 3%, it was added into the adjacent category. The three PCA-derived sleep dimensions and six CASD autism dimensions (i.e., problems with social interaction, perseveration, somatosensory disturbance, atypical communication, mood disturbance, and selective attention/fearlessness) were used as indicator variables to identify distinct patterns of sleep and CASD symptoms across different groups, while controlling for age, sex, IQ, and medication use. This approach provided a number of “clusters” or “latent variables” to which each subject belonged to. To determine the optimal number of latent classes in the present sample, we used five measures as follows: (a) Akaike’s information criterion (AIC), Bayesian information criterion (BIC), and sample-size adjusted Bayesian information criterion (sBIC). Lower values for these criteria indicate superior model fits; (b) Entropy: a measure of separation between latent classes (Celeux & Soromenho, 1996). A value close to 1 indicates a clear classification; (c) Lo–Mendell–Rubin adjusted likelihood ratio test (LMR-LRT) (Lo et al., 2001) and bootstrapped likelihood ratio test (BLRT) (McLachlan, 1987). The reference group (Class 1) was selected based on its stable characteristics across the indicator variables (i.e., sleep and CASD dimensions), making it a suitable baseline for interpreting differences among clusters. Three separate LCA’s were also conducted separating the sample into three age groups (1–4, 5–9, and 10–17 years old), and are reported as supplementary materials.

Third, multinomial logistic regression analysis (MNLRA) was used to identify demographic and clinical variables independently associated with the LCA-determined classes in comparison with the reference group. The magnitude of the association was expressed in odds ratios (OR) and 95% confidence intervals.

Finally, multivariable-adjusted general linear models (MGLM) were used to examine differences between the LCA-determined classes on internalizing symptoms, ADHD and objective neurocognitive testing, while controlling for age, sex, SES, race, medication use and IQ. In univariate analyses, categorical and continuous variables were analyzed with Chi-square test and analysis of variance, respectively.

## Results

### Sample Characteristics

The demographic and clinical characteristics of the sample are presented in Table 1. The sample of 1397 children and adolescents was predominantly white (88.5%), male (81.2%), had a parent with a non-professional occupation (65.9%) and were aged 6.1 ± 3.3years old with an IQ of 88.5 ± 27.2. About 29% of the sample reported use of medication (e.g., stimulant, mood stabilizer, antipsychotic, selective serotonin reuptake inhibitor, anticonvulsant). Prenatal complications were reported in 15.9% of the sample, difficult temperament during infancy in 23.6% and difficult temperament during pre-school ages in 62.3%.

**Table 1:** Demographic and clinical characteristics of the sample

	Total (*N* = 1397)	Class 1 (*n* = 765)	Class 2 (*n* = 367)	Class 3 (*n* = 203)	Class 4 (*n* = 62)	*P*-value
**Age**, years	6.1 ± 3.3	5.6 ± 2.9	7.5 ± 3.9	4.9 ± 2.4	9.3 ± 3.1	**< 0.01**
**Male** (%)	81.2%	80.0%	80.4%	87.7%	79.0%	0.084
**Medication use** (%)	28.7%	23.4%	30.0%	34.5%	67.7%	**< 0.001**
**Ethnic/racial minority** (%)	11.0%	10.5%	8.2%	18.2%	11.3%	**0.003**
**Non-professional SES** (%)	65.9%	67.7%	55.0%	77.3%	71.0%	**< 0.001**
**Presence of pregnancy complications** (%) ^a^	15.5%	13.8%	15.5%	18.3%	24.1%	0.449
**Difficult early childhood temperament**						
Infancy (%) ^b^	23.6%	17.6%	26.9%	31.3%	40.0%	**0.007**
Preschool (%) ^c^	62.3%	61.9%	53.8%	76.6%	72.4%	**0.012**
**Core Autism Symptoms**						
Social Interaction (Z-score)	0.00 ± 1.00	0.07 ± 0.98	-0.19 ± 0.99	0.00 ± 1.08	0.23 ± 0.77	**< 0.001**
Sum	4.39 ± 0.72	4.45 ± 0.71	4.25 ± 0.72	4.40 ± 0.78	4.56 ± 0.56	**< 0.001**
Perseveration (Z-score)	0.00 ± 1.00	0.01 ± 1.00	-0.17 ± 1.06	0.21 ± 0.86	0.08 ± 0.78	**< 0.001**
Sum	3.43 ± 0.71	3.44 ± 0.71	3.30 ± 0.76	3.58 ± 0.62	3.48 ± 0.56	**< 0.001**
Somatosensory Disturbance (Z-score)	0.00 ± 1.00	0.03 ± 1.00	-0.39 ± 0.90	0.52 ± 0.89	0.16 ± 0.86	**< 0.001**
Sum	5.50 ± 1.77	5.56 ± 1.78	4.81 ± 1.60	6.44 ± 1.59	5.79 ± 1.53	**< 0.001**
Communication (Z-score)	0.00 ± 1.00	0.12 ± 0.98	-0.22 ± 0.94	0.07 ± 1.03	-0.46 ± 0.98	**< 0.001**
Sum	3.56 ± 1.03	3.69 ± 1.01	3.33 ± 0.97	3.63 ± 1.07	3.08 ± 1.01	**< 0.001**
Mood (Z-score)	0.00 ± 1.00	-0.01 ± 0.97	-0.15 ± 1.07	0.23 ± 0.94	0.30 ± 0.78	**< 0.001**
Sum	2.70 ± 0.93	2.69 ± 0.91	2.56 ± 1.01	2.92 ± 0.88	2.98 ± 0.73	**< 0.001**
Selective Attention/Fearlessness (Z-score)	0.00 ± 1.00	0.58 ± 0.00	-1.63 ± 0.43	0.58 ± 0.00	0.58 ± 0.00	**< 0.001**
Sum	1.73 ± 0.47	2.00 ± 0.00	0.96 ± 0.20	2.00 ± 0.00	2.00 ± 0.00	**< 0.001**
**Sleep Factors** ^**d**^						
Disturbed Sleep	0.90 ± 0.78	0.66 ± 0.60	0.67 ± 0.64	1.79 ± 0.69	1.46 ± 0.79	**< 0.01**
Insufficient Sleep	0.85 ± 0.96	0.44 ± 0.53	0.58 ± 0.79	2.38 ± 0.53	1.03 ± 0.94	**< 0.01**
Hypersomnolence	0.30 ± 0.50	0.16 ± 0.28	0.35 ± 0.50	0.20 ± 0.34	1.67 ± 0.50	**< 0.01**
**Internalizing Symptoms** ^d^						
Anxiety	1.0 ± 0.72	0.83 ± 0.66	1.2 ± 0.7	1.1 ± 0.7	1.6 ± 0.6	**< 0.01**
Depression	0.41 ± 0.44	0.31 ± 0.35	0.41 ± 0.41	0.58 ± 0.57	0.86 ± 0.51	**< 0.01**
**Externalizing Symptoms** ^d^						
ADHD (*total score*)	1.7 ± 0.72	1.8 ± 0.6	1.3 ± 0.6	2.3 ± 0.5	2.0 ± 0.6	**< 0.01**
Inattention	1.9 ± 0.77	1.8 ± 0.7	1.6 ± 0.8	2.2 ± 0.6	2.31 ± 0.6	**< 0.001**
Impulsivity	1.7 ± 0.81	1.8 ± 0.7	1.2 ± 0.7	2.3 ± 0.5	2.21 ± 0.7	**< 0.001**
Hyperactivity	1.6 ± 0.97	1.7 ± 0.9	1.1 ± 0.9	2.3 ± 0.7	1.68 ± 0.9	**< 0.001**
**FSIQ**	88.5 ± 27.2	85.8 ± 28.0	93.9 ± 27.0	88.6 ± 24.4	89.6 ± 22.7	**< 0.01**
Working memory ^e^	87.4 ± 17.9	89.0 ± 18.6	88.2 ± 17.7	85.4 ± 14.7	80.8 ± 18.6	0.122
Processing speed ^f^	83.9 ± 16.8	85.9 ± 18.4	83.7 ± 14.9	84.5 ± 14.7	76.0 ± 17.0	**0.034**
**Achievement**						
Reading comprehension ^g^	96.0 ± 20.6	96.4 ± 21.9	97.9 ± 19.7	93.8 ± 18.0	89.4 ± 2.7	0.324
Written expression ^h^	85.8 ± 16.4	86.8 ± 17.5	88.4 ± 15.6	81.4 ± 11.6	77.4 ± 16.9	< 0.01*
Word reading ^i^	98.8 ± 19.7	100.2 ± 22.3	99.6 ± 17.2	99.5 ± 14.8	89.1 ± 20.4	**0.040**
Numerical operations ^i^	94.5 ± 19.9	96.7 ± 21.5	96.2 ± 18.5	92.6 ± 15.1	82.2 ± 19.0	< 0.01*

Note. Data are means ± SD. FSIQ = Full scale intelligence quotient. ^a^ 895 participants had missing data on pregnancy complications for a total sample of *N* = 502 (Class 1, *N* = 254, Class 2, *N* = 148, Class 3, *N* = 71, Class 4, *N* = 29). ^b^ 936 participants had missing data on infancy temperament for a total sample of *N* = 461 (Class 1, *N* = 233, Class 2, *N* = 134, Class 3, *N* = 64, Class 4, *N* = 30). ^c^ 941 participants had missing data on preschool temperament for a total sample of *N* = 456 (Class 1, *N* = 231, Class 2, *N* = 132, Class 3, *N* = 64, Class 4, *N* = 29). ^d^ 287 participants had missing PBS data for a total sample of *N* = 1110 (Class 1, *N* = 570, Class 2, *N* = 286, Class 3, *N* = 195, Class 4, *N* = 59). ^e^ 1105 participants had missing working memory data for a total sample of *N* = 292 (Class 1, *N* = 118, Class 2, *N* = 108, Class 3, *N* = 35, Class 4, *N* = 31). ^f^ 1097 participants had missing processing speed data for a total sample of *N* = 300 (Class 1, *N* = 121, Class 2, *N* = 111, Class 3, *N* = 37, Class 4, *N* = 31). ^g^ 1160 participants had missing reading comprehension data for a total sample of *N* = 237 (Class 1, *N* = 95, Class 2, *N* = 91, Class 3, *N* = 28, Class 4, *N* = 23). ^h^ 1157 participants had missing written expression data for a total sample of *N* = 240 (Class 1, *N* = 94, Class 2, *N* = 92, Class 3, *N* = 29, Class 4, *N* = 25). ^i^ 1095 participants had missing word reading & numerical operations data for a total sample of *N* = 302 (Class 1, *N* = 124, Class 2, *N* = 111, Class 3, *N* = 36, Class 4, *N* = 31)

### PCA

**Fig. 1:**
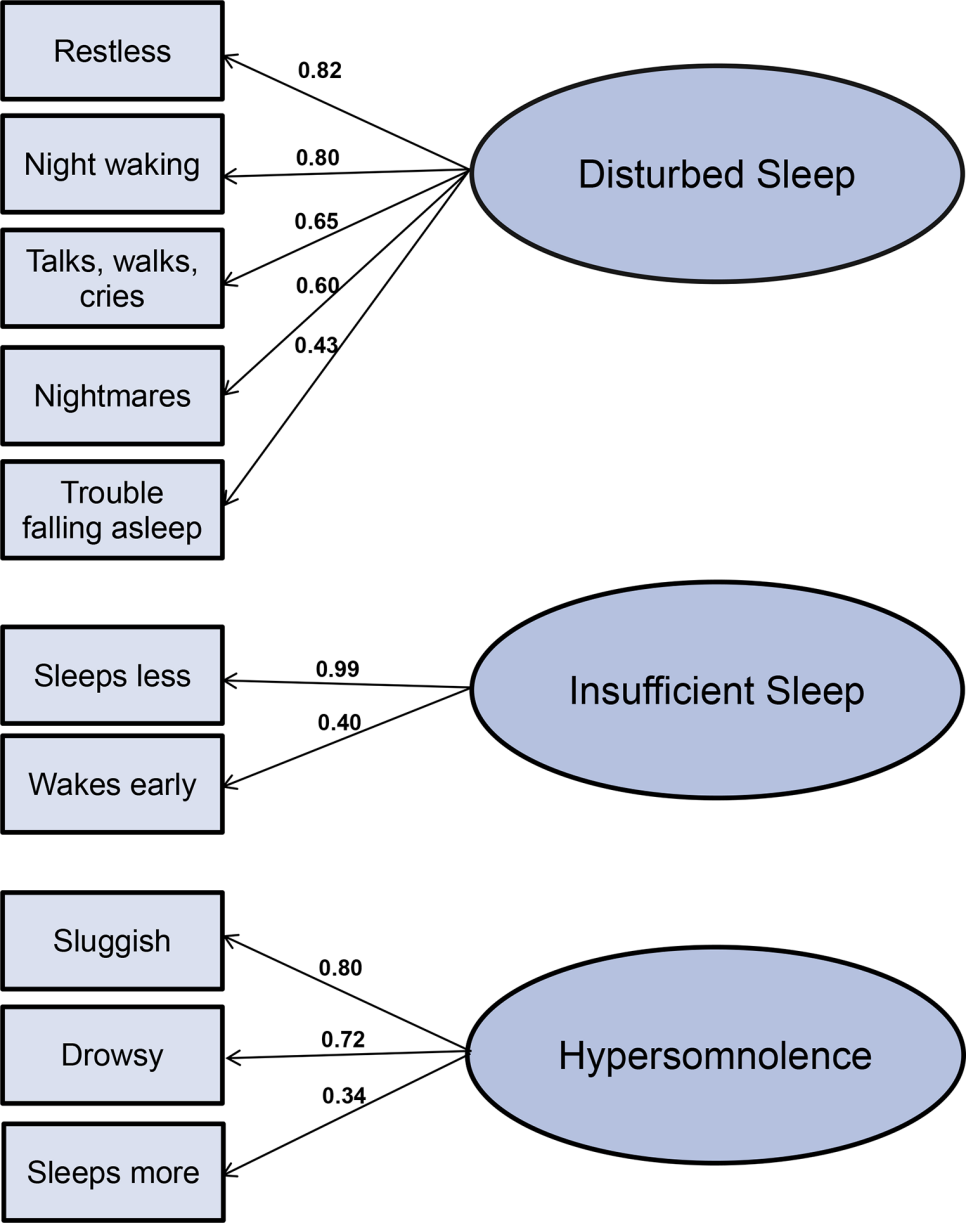
Principal Component Analysis (PCA) of 10 PBS items

Three clusters with distinguishable sleep phenotypes were retained from the EFA, that had eigenvalues > 1, with adequate goodness-of-fit (TLI = 0.91, RMSEA = 0.075); (a) disturbed sleep (trouble falling asleep, restless sleep, wakes often, nightmares, and talks/walks/or cries in sleep), (b) insufficient sleep (sleeps less and wakes early), and, (c) hypersomnolence (drowsy/sleepy, sluggish/slow moving, sleeps more) (see Fig. 1). The 3-component solution explained a total of 48.4% of the variance, with disturbed sleep contributing 23.5%, insufficient sleep contributing 12.2%, and hypersomnolence contributing 12.7%.

### LCA

**Fig. 2:**
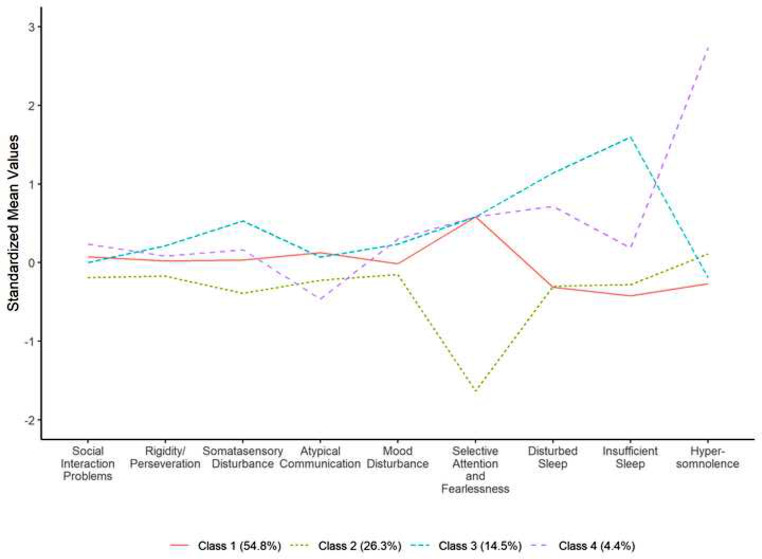
Latent Class Analysis (LCA) 4-Class Model

LCA revealed a 3-class and 4-class model to both have adequate fit (see Table 2). Specifically, based on IC measures, the 4-class model yielded better model fit, with acceptable entropy (i.e., 0.856). Although the LMR-LRT for the 4-class model was non-significant, the BLRT was significant, compared to the 3-class solution, which indicates the 4-class model was a better fit. Therefore, based on both conceptual grounds and considering all model fit statistics, the 4-class model was chosen, where Class 1 represented 54.8% (*n* = 765) of the sample, Class 2 26.3% (*n* = 367), Class 3 14.5% (*n* = 203), and Class 4 4.4% (*n* = 62). The standardized mean values on the three sleep dimensions and six CASD dimensions across four subgroups are plotted in Fig. 2.

**Table 2:** Comparisons of model fit indices and classification performance between 3-class and 4-class models

Measures	3-Class Model	4-Class Model
AIC	29164.844	28986.172
BIC	29594.694	29489.411
sBIC	29334.211	29184.456
Entropy	0.951	0.856
LMR-LRT	0.001	0.116
BLRT	0.000	0.000

Note: AIC: Akaike’s information criterion; BIC: Bayesian information criterion; sBIC: sample-size adjusted BIC; LMR-LRT: Lo-Mendell-Rubin adjusted likelihood ratio test; BLRT: bootstrap likelihood ratio test

Using Class 1 as the reference group, those in Class 2 (*n* = 367; 26.3%) were categorized by a similar degree of sleep problems as the reference group, but markedly less problems with ASD and ADHD symptomology, namely somatosensory disturbance, and higher IQ. Those in Class 3 (*n* = 203; 14.5%) were categorized by insufficient and disturbed sleep, younger in age, more likely to belong to a racial/ethnic minority, lower parental SES, difficult preschool temperament, somatosensory disturbance, internalizing symptoms (i.e., anxiety and depression), and ADHD symptoms (i.e., inattention, impulsivity, and hyperactivity). Those in Class 4 (*n* = 62; 4.4%) were categorized by hypersomnolence, being older, highest medication use, increased difficult infancy temperament, internalizing symptoms (i.e., anxiety and depression), ADHD symptomology and poorer objectively measured processing speed, and writing and math academic achievement (see Table 5).

*Age Stratification*. LCA were then conducted separately for each age group (1–4; *n* = 534, 5–9; *n* = 621, 10–17; *n* = 242).

*1–4 year olds.* The 3-class model yielded acceptable entropy (i.e., 0.893). While the LMR-LRT was non-significant, the BLRT was significant. Considering all model fit indices, the 3-class model was chosen. The standardized mean values on the three sleep dimensions and six CASD dimensions across the three subgroups were plotted in Figure S1. Using Class 1 (*N* = 383; 71.7%) as the reference group, Class 2 (*N* = 94; 17.6%) was categorized by markedly less problems with selective attention/ fearlessness, less somatosensory disturbance, less medication use, and female sex. Class 3 (*N* = 57; 10.7%) was categorized by more hypersomnolence.

*5–9 year olds.* The 2-class model yielded satisfactory entropy (i.e., 1.0). While the LMR-LRT was non-significant, the BLRT was significant. Considering all model fit indices, the 2-class model was chosen. The standardized mean values on the three sleep dimensions and six CASD dimensions between the two subgroups were plotted in Figure S2. Using Class 1 (*N* = 466; 74.9%) as the reference group, Class 2 (*N* = 155; 25.1%) was categorized by less problems with selective attention/fearlessness, higher IQ, and less medication use.

*10–17 year olds*. The 3-class model yielded satisfactory entropy (i.e., 0.996). While the LMR-LRT was non-significant, the BLRT was significant. Considering all model fit indices, the 3-class model was chosen. The standardized mean values on the three sleep dimensions and six CASD dimensions across the three subgroups were plotted in Figure S3. Using Class 2 (*N* = 104; 43%) as the reference group, Class 1 (*N* = 116; 48%) was categorized by less problems with selective attention/fearlessness. Class 3 (*N* = 22; 9%) was categorized by more disturbed and insufficient sleep and lower IQ.

### MNLRA

The multinomial logistic regression analysis (Tables 3 and 4), used for investigating the association of several independent variables with the LCA determined classes, revealed that Class 2 was significantly associated with older age, higher SES, difficult infant and preschool temperament, and Class 3 was significantly associated with male sex, younger age, minority status, lower SES, and difficult infant temperament, and Class 4 was significantly associated with older age and difficult infant temperament,.

**Table 3:** Sociodemographic outcomes

Variable	LCA Classes	OR	95% CI
Sex	Class 4	0.948	0.488–1.841
	Class 2	0.951	0.685–1.319
	Class 3	1.912*	1.208–3.026
Age	Class 4	1.338**	1.247–1.436
	Class 2	1.181**	1.136–1.227
	Class 3	0.915*	0.863–0.970
Race	Class 4	0.844	0.362–1.970
	Class 2	1.166	0.740–1.839
	Class 3	0.567*	0.368–0.874
Non-professional SES	Class 4	0.774	0.430–1.392
	Class 2	1.587**	1.217–2.069
	Class 3	0.654*	0.453–0.944

Note: *p ≤ 0.05; ** *p* ≤ 0.01

**Table 4:** Pregnancy and temperament outcomes

Variable	LCA Classes	OR	95% CI
Presence of pregnancy complications	Class 4	1.483	0.540–4.074
	Class 2	1.152	0.617–2.152
	Class 3	1.065	0.481–2.357
Difficult infant temperament	Class 4	3.373**	1.395–8.158
	Class 2	1.962*	1.128–3.412
	Class 3	1.932*	0.996–3.746
Difficult preschool temperament	Class 4	1.223	0.469–3.193
	Class 2	0.604*	0.379–0.963
	Class 3	1.783	0.903–3.520

Note: *p ≤ 0.05; ** *p* ≤ 0.01

### MGLM

*Internalizing and Externalizing Symptoms*. Compared to the reference group (Class 1), Class 2, 3, and 4, had significantly more anxiety symptoms (See Table 5). Compared to the reference group, Class 3 and 4, had significantly more depression symptoms, while Class 2 did not. Compared to the reference group, Class 3, and 4, had significantly more ADHD symptoms in terms of inattention and impulsivity. However, Class 3 had significantly more hyperactivity when compared to the reference group, but Class 2 did not.

**Table 5:** Outcomes across LCA-determined classes

	Class 1 (*n* = 765)	Class 2 (*n* = 367)	Class 3 (*n* = 203)	Class 4 (*n* = 62)	1 vs. 4	1 vs. 2	1 vs. 3
**Core Autism Symptoms**							
Social Interaction	0.08 ± 0.03	-0.20 ± 0.05	0.03 ± 0.07	0.08 ± 0.12	0.960	**< 0.001**	0.557
Perseveration	-0.01 ± 0.03	-0.08 ± 0.05	0.16 ± 0.07	0.17 ± 0.12	0.142	0.331	**0.021**
Somatosensory Disturbance	0.00 ± 0.03	-0.32 ± 0.05	0.49 ± 0.06	0.22 ± 0.12	0.090	**< 0.001**	**< 0.001**
Communication	0.07 ± 0.03	-0.12 ± 0.05	0.02 ± 0.06	-0.31 ± 0.12	**0.002**	**0.001**	0.475
Mood	0.00 ± 0.03	-0.16 ± 0.05	0.22 ± 0.07	0.18 ± 0.12	0.173	**0.012**	**0.005**
Attention/Fearlessness	0.58 ± 0.00	-1.64 ± 0.01	0.58 ± 0.01	0.57 ± 0.02	0.692	**< 0.001**	0.964
**Sleep Factors**							
Disturbed Sleep	0.66 ± 0.02	0.66 ± 0.03	1.7 ± 0.04	1.4 ± 0.08	**< 0.001**	0.995	**< 0.001**
Insufficient Sleep	0.44 ± 0.02	0.58 ± 0.03	2.3 ± 0.04	0.99 ± 0.08	**< 0.001**	**0.005**	**< 0.001**
Hypersomnolence	0.17 ± 0.01	0.31 ± 0.02	0.23 ± 0.02	1.5 ± 0.04	**< 0.001**	**< 0.001**	0.068
**Internalizing Symptoms**							
Anxiety	0.87 ± 0.02	1.1 ± 0.04	1.1 ± 0.04	1.4 ± 0.08	**< 0.001**	**< 0.001**	**< 0.001**
Depression	0.33 ± 0.01	0.37 ± 0.02	0.58 ± 0.03	0.75 ± 0.05	**< 0.001**	0.153	**< 0.001**
**Externalizing Symptoms**							
**ADHD** (*total score*)	1.8 ± 0.02	1.3 ± 0.03	2.2 ± 0.04	1.9 ± 0.08	**0.039**	**< 0.001**	**< 0.001**
Inattention	1.8 ± 0.03	1.6 ± 0.04	2.2 ± 0.05	2.1 ± 0.09	**0.006**	**< 0.001**	**< 0.001**
Impulsivity	1.8 ± 0.03	1.3 ± 0.04	2.2 ± 0.05	2.1 ± 0.09	**0.002**	**< 0.001**	**< 0.001**
Hyperactivity	1.7 ± 0.03	1.2 ± 0.05	2.2 ± 0.06	1.6 ± 0.11	0.682	**< 0.001**	**< 0.001**
**FSIQ**	86.5 ± 0.9	91.4 ± 1.4	91.1 ± 1.9	87.2 ± 3.4	0.844	**0.005**	**0.029**
Working memory	88.2 ± 1.5	88.1 ± 1.6	86.6 ± 2.9	83.2 ± 3.1	0.161	0.947	0.636
Processing speed	85.3 ± 1.5	83.5 ± 1.6	84.6 ± 2.8	77.4 ± 3.0	**0.022**	0.427	0.842
**Achievement**							
Reading comprehension	95.1 ± 1.6	98.5 ± 1.5	95.6 ± 2.9	95.6 ± 3.2	0.892	0.139	0.899
Written expression	87.8 ± 1.3	89.0 ± 1.3	82.0 ± 2.4	81.5 ± 2.7	**0.042**	0.527	**0.041**
Word reading	101.8 ± 1.5	100.5 ± 1.4	103.1 ± 2.7	98.6 ± 3.0	0.382	0.534	0.676
Numerical operations	99.3 ± 1.3	97.4 ± 1.2	94.0 ± 2.3	91.2 ± 2.6	**0.007**	0.306	**0.052**

Note. Data are means ± SEM adjusted for covariates (age, sex, SES, race, medication, IQ), except for Core Autism Symptoms, which are Z-scores. P-values are post-hoc comparisons from multivariable-adjusted linear models. Data are means ± SEM adjusted for covariates (age, gender, SES, race, medication) for working memory and processing speed) FSIQ = Full scale intelligence quotient

*Intelligence Quotient.* Compared to the reference group (Class 1), Class 2 and 3 had a significantly higher overall IQ score, while Class 4 had a similar overall IQ score. Class 2, 3, and 4 did not significantly differ on working memory from the reference group (See Table 5). Compared to the reference group (Class 1), Class 4 scored significantly lower on processing speed while Class 2 and 3 did not.

*Achievement*. Class 2, 3, and 4 did not significantly differ on reading comprehension or word reading from the reference group (Class 1) (See Table 5). Compared to the reference group, Class 3 and Class 4 scored significantly lower on written expression and numerical operations.

## Discussion

To our knowledge, this is the first study to address ASD heterogeneity by identifying latent classes or phenotypes based on both sleep dimensions and core autism symptoms in a large clinical sample of children and adolescents, as well as to explore differences in sociodemographic variables and functional outcomes between the resulting classes or phenotypes. The first key finding is that we identified three sleep dimensions (disturbed sleep, insufficient sleep and hypersomnolence) in autistic children and adolescents using PCA. The second key finding is that we identified four distinct phenotypic classes using LCA, which differed in their sleep profiles as well as in relation to clinical characteristics, demographics, internalizing/externalizing symptoms, and functional outcome variables; highlighting that ASD and sleep problems do not represent a uniform phenotype.

Our PCA results support the presence of three sleep dimensions among youth with ASD. Items pertaining to sleep disruption during the sleep period (e.g., “restless sleep”, “trouble falling asleep”, “night waking”) comprised the “disturbed sleep” factor. Items pertaining to inadequate amount of sleep or time in bed (e.g., “sleeps less than most other children”, “wakes up too early in the morning”) comprised the “insufficient sleep” factor. Items pertaining to excessive sleepiness (e.g., “drowsy or sleepy; not alert or awake”, “sleeps more than most other children”) comprised the “hypersomnolence” factor. These are important findings given the lack of existing studies delineating sleep dimensions within the ASD population. Previous studies have focused on dichotomizing sleep into “good/poor sleep” (Goldman et al., 2009) and “stable/unstable sleepers” (Cohen et al., 2017) instead of encompassing the full range of sleep problems autistic children and adolescents typically experience and the clustering of each single sleep symptom.

Our LCA results support the presence of four distinct phenotypic classes with observable sleep and care autism symptoms profiles. Using Class 1 as the reference group (54.8%), youth in Class 2 (26.3%) are characterized by a similar degree of sleep problems but higher overall cognitive functioning, and less problems with ASD (i.e., limited social skills, perseveration, somatosensory disturbance, mood and selective attention) and ADHD (i.e., inattention, impulsivity and distractibility) symptomology. When examining item level data for the selective attention factor, the low level is driven by the lack of endorsement of the “limited safety awareness/ fearlessness/ oblivious to danger” item, as opposed to the “selective attention/hyper focus” item, perhaps explained by older age and higher functioning individuals in this phenotype. Furthermore, youth in Class 2 show similar levels of depressive symptoms and higher levels of anxiety compared to the reference group.

Youth in Class 3 (14.5%) were characterized by the presence of insufficient and disturbed sleep, being younger, minority, lower SES, difficult preschool temperament, more problems with somatosensory disturbance, and hyperactivity, when compared to Class 1. The observed relationship between sensory over responsivity and sleep problems in ASD as suggested by previous studies (Mazurek et al., 2015; Mazurek et al., 2019), aligns with the characteristics of Class 3, particularly their prevalence of insufficient and disturbed sleep and high somatosensory disturbance. It is plausible that symptoms such as somatosensory disturbance, difficult temperament, and ADHD-related hyperactivity can all contribute negatively to sleep patterns in a synergistic manner. For instance, autistic individuals often exhibit heightened sensitivity to environmental stimuli and challenges in regulating arousal, potentially leading to various sleep disturbances including bedtime resistance, prolonged sleep onset latency, frequent night awakenings, short sleep duration, parasomnias, and early morning awakenings. These factors may manifest independently or in a comorbid manner.

Compared to Class 1, youth in Class 4 (4.4%) are characterized by the presence of hypersomnolence as well as being older, higher medication use, difficult infant temperament, internalizing symptoms, inattentiveness and poorer objectively-measured processing speed, writing achievement and math achievement. The co-occurrence of hypersomnolence with internalizing symptoms is consistent with previous research documenting the comorbidity of hypersomnia and depression among youth with ASD, particularly adolescents (Mayes et al., 2009a, b; Mazurek & Petroski, 2015). Previous research exploring the role of age on sleep problems in youth with ASD found that daytime sleepiness was a problem for 39% of adolescents with ASD (Mayes et al., 2022). Together with the current study, these results are consistent with developmental changes in the circadian rhythm following puberty that can result in delayed sleep onset and increased need for sleep (Carskadon, 2011), as well as environmental, motivational and social factors that contribute to daytime sleepiness in adolescence. Of note, 67.7% of youth in Class 4 were utilizing medication, which is consistent with previous studies identifying a linear trend of age and medication utilization for autistic youth (Oswald & Sonenklar, 2007). Results further showed evidence for the association of this phenotype on an objectively measured area of executive functioning, namely processing speed and written expression, and numerical operations achievement and parent-reported inattention. Our findings are consistent with previous studies suggesting that hypersomnolence is associated with attention and learning problems and poorer performance in processing speed and working memory (Anderson et al., 2009; Beebe, 2011; Calhoun et al., 2012).

Furthermore, previous research utilized socio-ecological and systems frameworks to disentangle the etiology of sleep patterns and problems in youth (Meltzer et al., 2021). Considering this social-ecological framework, factors at multiple socio-ecological levels likely interact in nuanced and complex ways to influence pediatric sleep over time. For example, social and health disparities for minority groups and those with lower SES often precipitate and perpetuate the onset and persistence of sleep problems for children in the general population, and maybe especially true for youth with ASD. Moreover, autistic individuals from marginalized communities may face additional barriers to accessing adequate healthcare and support services for addressing sleep problems. These disparities can result in delayed diagnosis and intervention, leading to poorer health outcomes and reduced quality of life. The demographic and clinical presentation of Class 3 indicates that early behavioral sleep interventions for insufficient and disturbed sleep as well as parent management training to address difficult temperament and ADHD symptoms could be especially beneficial for this group to mitigate cascading adverse health outcomes.

Based on the findings above, we concluded that a four-class phenotypic model of children with ASD best describes our data and improves phenotypic characterization of autistic children. The identification of these classes provides a unique contribution to the literature, given that no studies have examined classes based on core autism symptoms as well as detailed sleep factors. The differences in the patterns among the classes in children with ASD highlight the importance of sleep in the phenotypic characterization of autistic children. It is interesting to note that all core autism symptoms with the exception of selective attention/fearlessness were similarly, frequently reported for youth in each latent class, suggesting that sleep problems are in fact the unique identifiers driving these phenotypic findings.

Finally, age restricted subsets identified differences in phenotypic groups. Among children with ASD 1–4 years old, there was a class characterized by more hypersomnolence (10.7%), which is inconsistent with what is expected for this age group. However, it is possible that children in that class had an undiagnosed, organic sleep disorder (i.e., sleep disordered breathing, restless legs syndrome, periodic limb movement disorder) impacting their sleep quality and thus their sleepiness throughout the day. Further, the prevalence of hypersomnolence is consistent with prevalence rates in the autistic population (Kolla et al., 2019). Among autistic children 5–9 years old, the 2-class model identified a class (25.1%) characterized by significantly less selective attention/fearlessness and similar profiles in terms of other core autism symptoms and sleep problems. Among children and adolescents 10–17 years old with ASD, about half of the group was characterized by less selective attention/fearlessness. Additionally, it is evident that the insufficient sleep factor is primarily driven by the 10–17 year age group. It is interesting to note that insufficient sleep is likely not contributing to hypersomnolence. This finding is consistent with previous literature documenting the prevalence of insufficient sleep in adolescence and recognizing insufficient sleep as a serious health risk among this age group. Lastly, the age analyses consistently identified a class characterized by significantly less selective attention/fearlessness and similar profiles in terms of other core autism symptoms and sleep problems, which is similar to Class 2 in the overall LCA. Of note, this pattern is not driven by a certain age group and continues to be driven by the absence of parent-endorsed fearlessness.

The findings from the current study carry implications for clinical practice and additional areas for future research. Creating phenotypic subgroups of autistic children will help elucidate the various etiologies that contribute to ASD symptomology and functional impairment. The four-class model of ASD described herein is a first step in understanding how sleep problems associated with ASD cluster together across childhood and adolescence, and can serve as a foundation for the development of enhanced diagnostic algorithms in future research and clinical practice. Furthermore, these symptom profiles can be used to guide screening and diagnostic efforts and the appropriate selection and tailoring of strategies to address specific sleep problems. For example, one class of children with ASD clearly had hypersomnolence. It is therefore important for healthcare providers to be aware of these different presentations in autistic children and adolescents as they may benefit from varying clinical interventions based on their specific sleep-arousal problem. Since sleep problems were the unique identifiers driving these phenotypic findings, interventions adapted for the autistic population targeting modifiable behaviors, such as sleep, are essential in improving clinical and functional outcomes. Furthermore, future research should identify which subgroups of children with ASD are most likely to benefit from specific intervention options, and to better understand the varying etiological pathways relevant to autism. Future studies could further explore the independent effects of other sociodemographic factors such as household income, parent educational level, specific minority populations, and intellectual disability status on phenotypic subgroups of autistic children. Finally, as the findings indicate there is an association with core autism symptoms and sleep problems in youth with ASD, it is imperative for future studies to identify the causal mechanisms that may explain these complex relationships.

Some limitations of our study should be noted. This was a cross-sectional study and, therefore, we could not assess causality. Furthermore, generalization of results may be limited by the homogeneity of demographics in the sample (i.e., predominantly Caucasian, non-ethnic/racial minority, male). Replication studies are needed to demonstrate the stability of out phenotypes in other samples of children and longitudinal studies are needed to examine the trajectory of phenotypes over time. Moreover, we relied on parent-reported symptoms of sleep problems rather than self-reported data in adolescence, and we do not know whether individuals at older ages experienced more sleep problems than was reported. The definition of sleep problems was that of symptoms rather than a disorder or syndrome, as it did not include general diagnostic criteria International Classification of Sleep Disorders-Third Edition (ICSD-3) nor did we exclude other highly prevalent sleep disorders (e.g., delayed sleep phase disorder, sleep disordered breathing) due to lack of objective measures of sleep (e.g., polysomnography, actigraphy). Having diagnostic sleep criteria could further elucidate the cause of the sleep problems present in the different classes (i.e., sleep disordered breathing contributing to elevated hypersomnolence) and provide a more fine-grained understanding of the phenotypes identified in the current study. Additionally, different IQ tests were used to cover the mental age range of the children in the study, and the presence of intellectual developmental disability per se (i.e., IQ and adaptive functioning) was not included in the sample. Notwithstanding, the current study has several strengths. A uniqueness of this dataset is the size and breadth of information included given the thorough evaluations that were conducted. Additionally, the wide age range of 1 to 17 years of age for children and adolescents with ASD is a significant strength, as it encompasses several critical developmental periods. Furthermore, diagnoses were based on thorough evaluations (i.e., parent-report, teacher report, interviews with the clinician, review of medical records, clinical observations, and psychological testing) rather than a diagnosis solely from a physician or school professional.

Our study identified 4 classes that were distinguished on differing profiles in relation to core autism symptoms and associated sleep problems. Our findings highlight the importance of including sleep variables in the phenotyping of autistic children and adolescence, given that sleep problems are in fact the unique identifiers driving these phenotypic findings. Further replication studies are warranted for validation of the subgroups identified in these analyses.

## Electronic supplementary material

Below is the link to the electronic supplementary material.

Supplementary Material 1

Supplementary Material 2

Supplementary Material 3

Supplementary Material 4
