# Supplementary figures and images for: Autism Spectrum Disorder Phenotypes Based on Sleep Dimensions and Core Autism Symptoms
Source: J Autism Dev Disord. 2025 Apr 17;55(12):4412–24. doi: 10.1007/s10803-025-06822-y (PMC12589355; doi:10.1007/s10803-025-06822-y)

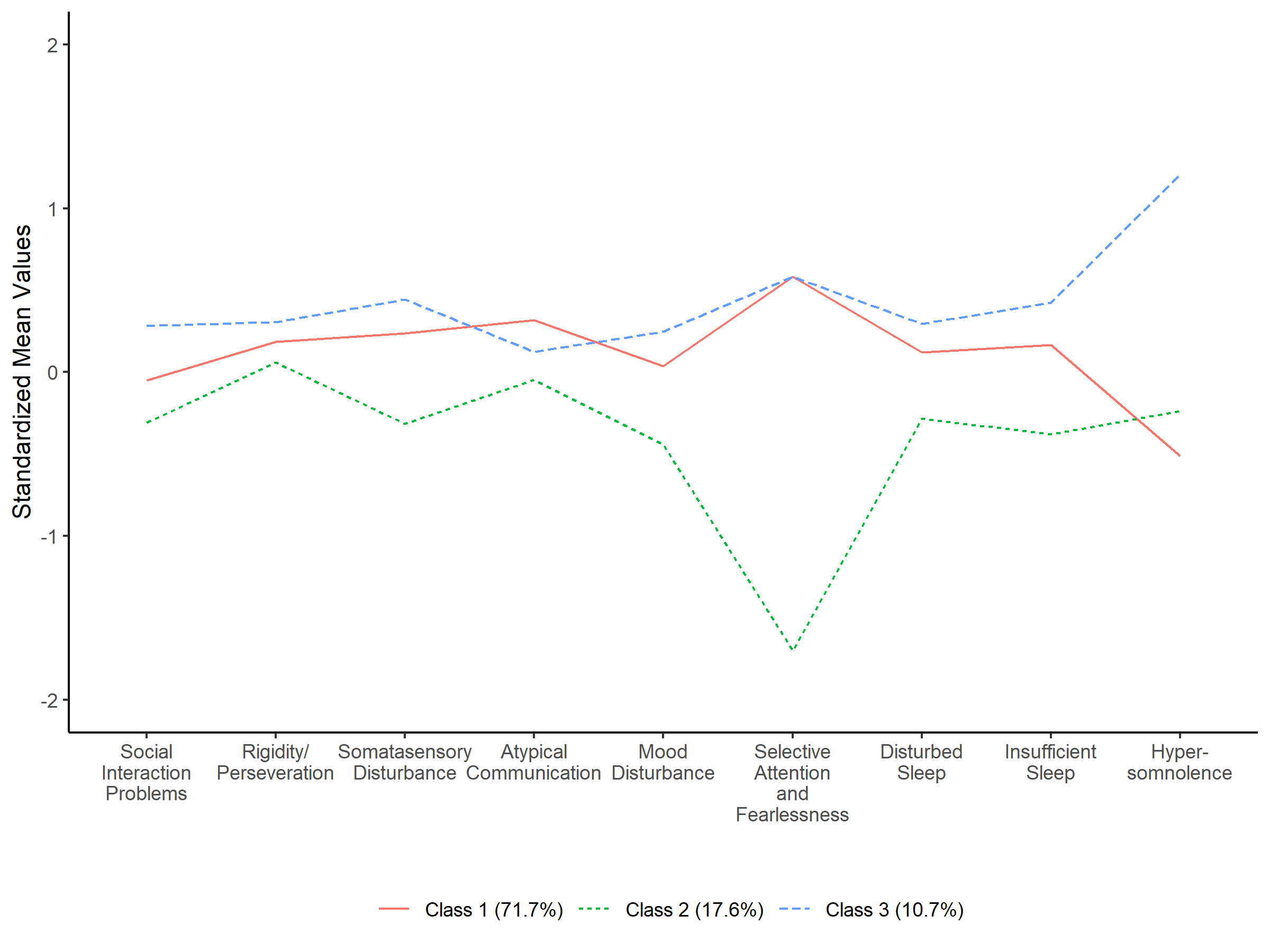

Supplement: Supplementary file 1 — Supplementary Material 1 [file 10803_2025_6822_MOESM1_ESM.tiff]

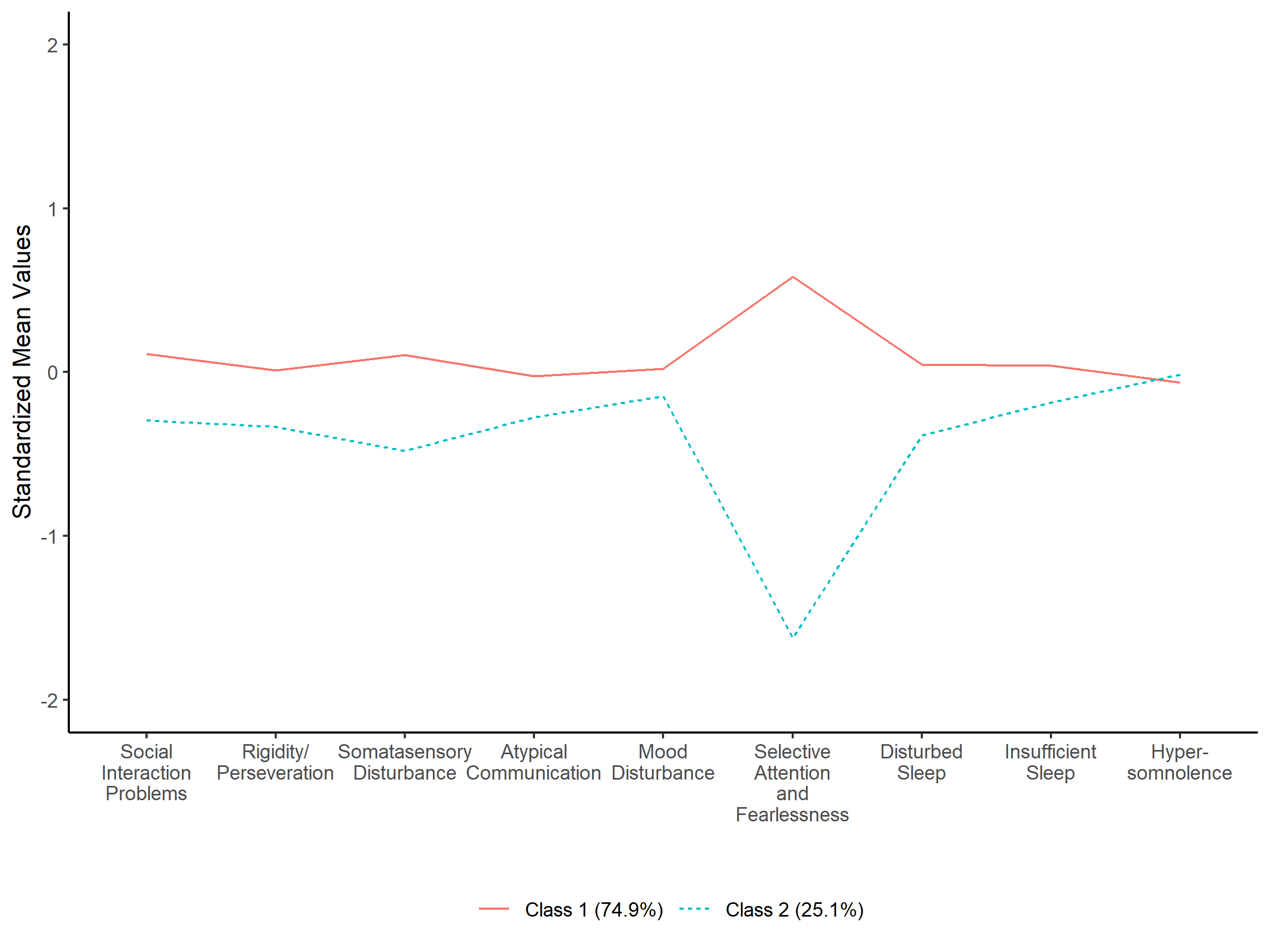

Supplement: Supplementary file 2 — Supplementary Material 2 [file 10803_2025_6822_MOESM2_ESM.tiff]

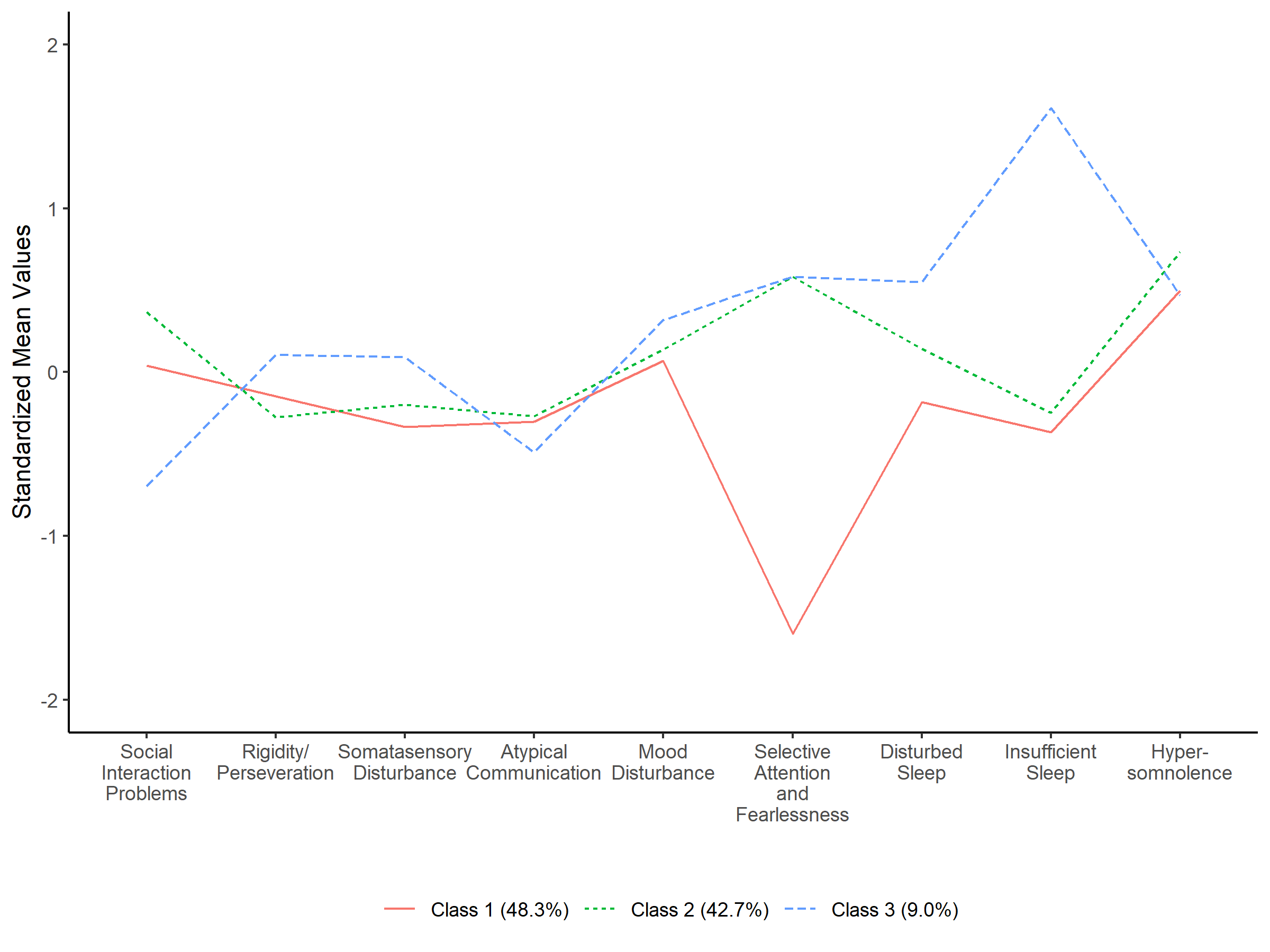

Supplement: Supplementary file 3 — Supplementary Material 3 [file 10803_2025_6822_MOESM3_ESM.tiff]
