# Supplementary material for: Autism Spectrum Disorder Phenotypes Based on Sleep Dimensions and Core Autism Symptoms
Source: J Autism Dev Disord. 2025 Apr 17;55(12):4412–24. doi: 10.1007/s10803-025-06822-y (PMC12589355; doi:10.1007/s10803-025-06822-y)
Supplement: Supplementary file 4 — Supplementary Material 4 [file 10803_2025_6822_MOESM4_ESM.docx]

**Supplemental Table 1.** Model fit indices (age group: 1-4 years old)

| **Measures** | **3-Class Model** |
| --- | --- |
| AIC | 8816.321 |
| BIC | 9158.752 |
| sBIC | 8904.807 |
| Entropy | 0.893 |
| LMR-LRT | 0.765 |
| BLRT | 0.000 |

Note: AIC: Akaike’s information criterion; BIC: Bayesian information criterion; sBIC: sample-size adjusted BIC; LMR-LRT: Lo-Mendell-Rubin adjusted likelihood ratio test; BLRT: bootstrap likelihood ratio test.

**Supplemental Table 2.** Model fit indices (age group: 5-9 years old)

| **Measures** | **2-Class Model** |
| --- | --- |
| AIC | 11324.561 |
| BIC | 11621.460 |
| sBIC | 11408.746 |
| Entropy | 1 |
| LMR-LRT | 0.491 |
| BLRT | 0.000 |

Note: AIC: Akaike’s information criterion; BIC: Bayesian information criterion; sBIC: sample-size adjusted BIC; LMR-LRT: Lo-Mendell-Rubin adjusted likelihood ratio test; BLRT: bootstrap likelihood ratio test.

**Supplemental Table 3.** Model fit indices (age group: 10-17 years old)

| **Measures** | **3-Class Model** |
| --- | --- |
| AIC | 4306.501 |
| BIC | 4585.616 |
| sBIC | 4332.030 |
| Entropy | 0.996 |
| LMR-LRT | 0.627 |
| BLRT | 0.000 |

 Note: AIC: Akaike’s information criterion; BIC: Bayesian information criterion; sBIC: sample-size adjusted BIC; LMR-LRT: Lo-Mendell-Rubin adjusted likelihood ratio test; BLRT: bootstrap likelihood ratio test.
